# FGF8 Activates Proliferation and Migration in Mouse Post-Natal Oligodendrocyte Progenitor Cells

**DOI:** 10.1371/journal.pone.0108241

**Published:** 2014-09-26

**Authors:** Pablo Cruz-Martinez, Almudena Martinez-Ferre, Jesus Jaramillo-Merchán, Alicia Estirado, Salvador Martinez, Jonathan Jones

**Affiliations:** 1 Neuroscience Institute, University Miguel Hernández (UMH-CSIC), San Juan, Alicante, Spain; 2 IMIB-Hospital Universitario Virgen de la Arrixaca, Universidad de Murcia, Murcia, Spain; Hospital Nacional de Parapléjicos - SESCAM, Spain

## Abstract

Fibroblast growth factor 8 (FGF8) is a key molecular signal that is necessary for early embryonic development of the central nervous system, quickly disappearing past this point. It is known to be one of the primary morphogenetic signals required for cell fate and survival processes in structures such as the cerebellum, telencephalic and isthmic organizers, while its absence causes severe abnormalities in the nervous system and the embryo usually dies in early stages of development. In this work, we have observed a new possible therapeutic role for this factor in demyelinating disorders, such as leukodystrophy or multiple sclerosis. In vitro, oligodendrocyte progenitor cells were cultured with differentiating medium and in the presence of FGF8. Differentiation and proliferation studies were performed by immunocytochemistry and PCR. Also, migration studies were performed in matrigel cultures, where oligodendrocyte progenitor cells were placed at a certain distance of a FGF8-soaked heparin bead. The results showed that both migration and proliferation was induced by FGF8. Furthermore, a similar effect was observed in an in vivo demyelinating mouse model, where oligodendrocyte progenitor cells were observed migrating towards the FGF8-soaked heparin beads where they were grafted. In conclusion, the results shown here demonstrate that FGF8 is a novel factor to induce oligodendrocyte progenitor cell activation, migration and proliferation in vitro, which can be extrapolated in vivo in demyelinated animal models.

## Introduction

Oligodendrocyte degeneration and subsequent myelin loss is the primary cause of multiple sclerosis and leukodistrophy, among other demyelinating conditions. This may be due to either an autoimmune attack (multiple sclerosis) or metabolic/genetic defects (leukodistrophy) [1]–[3]. Myelin loss causes irreversible neurological deficits, as the oligodendrocytes are crucial both for the metabolic support of the axons [4] as well as the correct transmission of the nerve impulse. Thus, oligodendrocyte loss implicates neuronal degeneration.

Oligodendrocyte progenitor cells (OPCs) are located throughout the central nervous system, which can be detected by the expression of the proteoglycan NG2 [5], [6]. These cells, after an acute demyelinating lesion, are activated and differentiate into mature oligodendrocytes as early as 7 days after the injury [7]. Depending on the type of demyelinating lesions, multiple sclerosis is divided into two phases: acute and chronic. In the acute phase, the nearby OPCs invade the lesion and remyelinate [8], [9], while in the chronic phase the migratory and differentiating mechanisms of the progenitors are affected, resulting in sustained and progressive demyelination [10]. This latter phase is partly due to the lack of factors that stimulate regeneration and/or to the presence in the lesion of molecules that inhibit remyelination [11]. In this case, the stimulation of OPCs to migrate and differentiate by external sources is a viable therapeutic option in order to favor neuronal survival [12].

Previous works in our lab have proven that OPCs may be activated and remyelination induced using bone marrow stem cells [13]. This was due to the secretion of certain soluble factors. In this work, we analyzed the effect that fibroblast growth factor 8 (FGF8) may exert on the activation and differentiation of OPCs. Fibroblast growth factors (FGFs) are a family of soluble protein ligands that play numerous roles during embryonic development, tissue homeostasis and metabolism. There are 22 known members, with different receptor binding affinities and biological functions [14]–[16]. Depending on the type and receptor, FGFs activate the RAS-MAPK or PI3K-AKT pathway, promoting proliferation, survival and/or motility in various cell types, including oligodendrocytes [17]–[23].

Of the FGFs members, FGF8 is known to be implicated in early vertebrate brain patterning, and its inhibition causes early embryonic death with absence of the entire mesencephalic and cerebellar primordia [24]–[28], as well as important thalamic and telencephalic malformations [29], [30]. FGF8 is capable of binding to all 4 FGF receptors, with different affinities among them [31], [32]. When the growth factor binds to its receptor, phosphorylation of the Extracellular signal Regulated Kinase 1/2 (ERK1/2) usually occurs, activating the RAS-MAPK intracellular pathway [33]–[36].

The aim of this study is to analyze if FGF8 may exert an effect on post-natal OPCs, both in vitro and in vivo. As this factor is a known morphogen during embryonic development, the rationale is that FGF8 may be used to induce the mobilization, proliferation, and differentiation of OPCs, as well as possibly remyelination. The results of this work may indicate a possible use for this morphogen in therapies to induce remyelination such as in multiple sclerosis.

## Materials and Methods

### 1 Animal experimentation

All the experiments with animals have been performed in compliance with the Spanish and European Union laws on animal care in experimentation (Council Directive 86/609/EEC), and have been analyzed and approved by the Animal Experimentation Committee of the University Miguel Hernandez and Neuroscience Institute, Alicante, Spain (Reference IN-SM-001-12). All efforts were made to minimize suffering. Mice were bred and maintained in our animal facilities.

### 2 Oligodendrocyte progenitor cell isolation and culture

The protocol used is similar to previous publications [37]–[39]. Briefly, the forebrains of newborn mice were removed, diced into 1 mm fragments, digested at 37°C for 20 min with a collagenase (1 mg/ml)/dispase (3 mg/ml) solution and mechanically dissociated. The resulting cellular suspension was then placed on poly-L-lysine coated dishes with the following culture medium: DMEM, 15% FBS, 1% penicillin/streptomycin. After 7–10 days, the culture consisted mainly in astrocytes with a small number of oligodendrocyte progenitor cells (OPCs) growing on top. In order to purify the culture, the cells were trypsinized with 0.05% trypsin and shaken to separate the OPCs from the astrocytes. Cell suspensions were then plated onto uncoated Petri dishes for 1 h to further remove residual contaminating microglia/astrocytes. The cell suspension was then replated in DMEM with 15% FBS, 1% penicillin/streptomycin, 10 ng/ml PDGF-AA and 10 ng/ml FGF2 for 7 days to stimulate the proliferation of OPCs. This resulted in a cell culture of mainly OPCs. Experiments were performed in triplicate (i.e. isolating OPCs from 3 different mice), and a total of 4 independent experiments were performed.

### 3 Brdu/Proliferation Analysis, Immunocytochemistry and Immunohistochemistry

A standard immunocytochemistry protocol was used. Twenty-four hours before fixing the cells, 1 µg/ml of BrdU was added to the culture to allow its incorporation. Primary antibodies used were anti-BrdU (1∶200, Dakocytomation, Denmark), anti-NG2 (1∶150, Chemicon), anti-PDGFR-α (1∶200, Santa Cruz Biotechnology), anti-Olig2 (1∶500, Santa Cruz Biotechnology), anti-O4 (1∶1000, Chemicon), anti-MBP (1∶100, Oncogene Research Products), and DAPI as nuclear staining. As for secondary antibodies, Alexa Green (1∶500, Molecular Probes) was used to stain in green, while for red staining a biotinylated secondary antibody was used (1∶200, Vector Laboratories, Burmingham, California) followed by an incubation with streptavidin conjugated with Cy3 (1∶500). Samples were visualized and images taken with a Leica fluorescence microscope (Leica DMR, Leica Microsystems).

For immunohistochemical analysis of the in vivo experiments, the mice were anesthetized and perfused using 4% filtered paraformaldehyde (PFA) in phosphate buffer (pH 7.4). The brain was carefully excised and kept in 4% PFA overnight. After fixation, the samples were cryoprotected for 12 hours at 4°C in 15% sucrose, followed by incubation overnight in 30% sucrose. Finally, samples were embedded in Neg −50 Frozen Section Medium (Richard-Allan Scientific, Kalamazoo, Michigan). Twelve micrometer transverse or longitudinal serial sections were obtained using a Microm HM525 cryostat and mounted on seven parallel series and processed for immunohistochemistry. Histological samples were observed under a fluorescence microscope (Leica DM6000D, Leica Microsystems). Micrographs were taken with DFC350/FX and DC500 Leica cameras.

### 4 Quantitative Real Time PCR

Total mRNA of the cells was isolated using the Trizol protocol (Invitrogen). A total of 5 micrograms of mRNA was reverse-transcribed, and approximately 100 ng of cDNA was amplified by Real Time PCR using Power SYBR Green Master mix (Applied Biosystems). All the samples were run in triplicate using the StepOne Plus Real-Time PCR system (Applied Biosystems) and analyzed with the StepOne Software. Analyses were carried out using the delta C(T) method and calculated relative to GAPDH (forward: AGGTCGGTGTGAACGGATTTG, reverse: GGGGTCGTTGATGGCAACA). The results were normalized with respect to the control condition, which presented a value of 1, using the same approach as in our previous report [40]. The following primers were used: PDGFR-α (Forward: TCCATGCTAGACTCAGAAGTCA, Reverse: TCCCGGTGGACACAATTTTTC), NG2 (Forward: GGGCTGTGCTGTCTGTTGA, Reverse: TGATTCCCTTCAGGTAAGGCA), Olig2 (Forward: CTAATTCACATTCGGAAG, Reverse: AAAAGATCATCGGGTTCT).

### 5 Matrigel Cultures

The method used was similar to that previously published [41]. A total of 20,000 oligodendrocyte progenitor cells were re-suspended in 5 µl of D-MEM and 20 µl of matrigel basement membrane matrix (BD Bioscience, 10 ml). D-MEM supplemented with 15% FBS was used as culture medium, as in the differentiation protocol of oligodendrocyte progenitor cells. The re-suspended cells were spread on a tissue culture dish (Becton Dickinson Labware), solidified during 25 min at 37°C and culture medium was added. Afterwards the cell-matrigel was cut in small squares of 125 µm^2^ and put on a solidified matrigel drop. Heparin acrylic beads (Sigma) were first rinsed in phosphate-buffered saline (PBS) four to six times and then soaked in FGF8 solution (5000 ng/ml; R&D) at 4°C overnight. The beads were then rinsed three times in PBS and put on the solidified matrigel drop 500 µm from the place where cells-matrigel was located. Control beads were soaked only in PBS and implanted in the same manner. FGF inhibitor (SU5402) was diluted in culture medium to a final concentration of 20 µM and soaked in Affigel blue beads (Bio-Rad), using the same procedure as described for FGF8-soaked beads. Then, 25 ml of a 1∶1 matrigel:culture medium mix was added, covering the drop where cells and beads were placed. After 2 h at 37°C, culture medium was added. Finally, 24 h and 48 h afterwards, the migrating and/or non-migrating-cells were observed under a dissecting microscope.

To confirm an FGF8-mediated attracting effect, the proximal/distal rate was calculated in control and FGF8-embedded cultures. This was performed similar to a previous publication [42]. Briefly, the images of the cell clusters were analyzed by imaging software to draw a 45° arc from the cluster towards the beads. The number of cells counted inside this arc (proximal cells) was divided by the number of cells outside the arc (distal cells), obtaining a proximal/distal rate. Values greater or less than 1 indicated more or less cells, respectively, near the bead.

### 6 Cuprizone Treatment of Mice as an In Vivo Chronic Demyelinating Model

The approach used was similar to our previously published report [13]. Briefly, Four-week old C3H/He mice were given a diet with 0.2% cuprizone (w/w) for 12 weeks ad libitum in order to obtain a chronic version of toxicity-induced demyelination, but with the following changes. Finely-powdered cuprizone (Sigma-Aldrich, St. Louis, MO) was dissolved in 60% tap water (w/v) with 0.5% (w/v) commercialized cane sugar. The cane sugar was added to avoid the weight loss observed in other studies due to the cuprizone treatment. After 12 weeks, the mice were grafted with the FGF8-soaked beads (or culture medium in the case of sham operated group) and returned to normal chow until their sacrifice.

### 7 FGF8-soaked heparin bead transplantation

The surgical procedure and coordinates used to inject the FGF8-heparin beads was the same as our previous report [13]. Before the surgical procedure, 0.1 mg/kg of buprenorphine (Buprex, Schering-Plough, Madrid, Spain) was injected into the mice. Isoflurane (Esteve Veterinary, Milan, Italy), an inhalational anesthesia, was used, and the mice were placed on a stereotaxic apparatus (Stoelting, Wheat Lane Wood Dale, IN, USA). The animals were monitored, and anesthesia concentration controlled. After the injection, the incision was sutured and the mice were monitored throughout the whole process of experimentation. A total of 8 cuprizone-treated mice were operated, 4 with FGF8-embedded heparin beads (at 1 mg/ml) and 4 sham controls (where PBS-soaked beads were used). After 1 month, the mice were sacrificed.

To quantify NG2 and MBP immunoreactivity, a similar approach as published in [13] was used. Briefly, images were taken and changed to an 8-bit black and white image. Then, the percentage of illuminated pixels was calculated, and compared between the experimental groups.

### 8 Statistical Analysis

Statistical significance between control and experimental groups were calculated with Sigmaplot v12.0 software, using the paired t-test and one-way ANOVA test where applicable, establishing the level of significance at p<0.05. Values are measured as mean +/− standard deviation.

## Results

### 1 FGF8 Increases Proliferation in Cultured Oligodendrocyte Progenitor Cells (OPCs)

**Figure 1:**
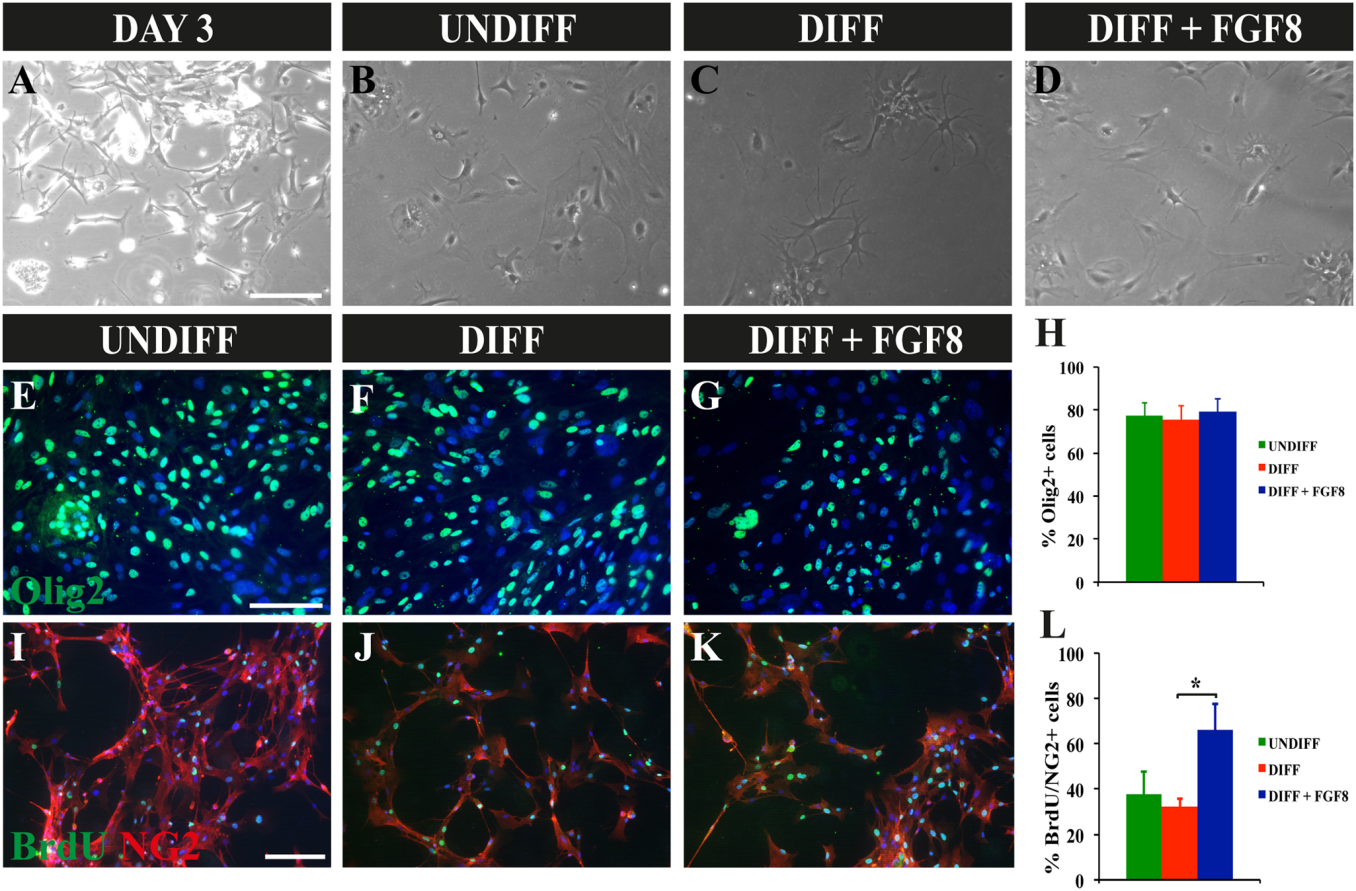
Culture and Differentiation of Oligodendrocyte Progenitor Cells (OPCs). A) OPCs cultured for 3 days after extraction on poly-L-lysine coated culture dishes. Numerous astrocytes are present. B) OPCs after passaging and expanding in the presence of FGF2 and PDGF-AA, to induce proliferation and maintenance of the OPCs. C) OPCs cultured in differentiation inducing medium for 7 days. Mature oligodendrocytes appear in the culture. D) OPCs cultured for 7 days in the differentiating medium and FGF8. All images taken at 100X magnification. E–G) Immunocytochemistry of OPCs in undifferentiating medium (E), differentiation inducing medium (F), and in the presence of FGF8 (G), staining for Olig2 (in green) and DAPI for nuclear staining (in blue). H) Histogram presenting the percentage of Olig2+ cells in the various cultures observed in E–G. Percentages are with respect to the total number of cells, calculated by DAPI staining. I–K) Immunocytochemistry of NG2+/Brdu+ cell in undifferentiating medium (I), differentiation inducing medium (J), and in the presence of FGF8 (K). L) Histogram presenting the percentage of NG2+/BrdU+ cells in the different cultures, with respect to the total number of cells. *p<0.05 (paired t-test), n = 4. Scale Bar indicates 150 µm for A–D and 100 µm for E–G and I–K.

The OPCs were isolated from P0–P2 newborn mice and cultured on poly-L-lysine coated culture dishes. The initial culture was a mixture of astrocytes, OPCs and other cell types, the OPCs being mainly detected on top of the astrocytes (Figure 1A). After 7 days of culture, the cells were treated with trypsin and shaken in order to detach the OPCs and re-plated onto new culture dishes, where they were further expanded using mitogens PDGF-AA and FGF2 (Figure 1B). In this fashion, after an additional 7 days of culture the majority of the cells detected corresponded to OPCs, as indicated in previous publications [35]–[37].

In order to induce OPCs differentiation, the culture medium was changed removing the mitogens and adding fetal bovine serum, resulting in spontaneous differentiation (Figure 1C). Mature oligodendrocytes could be detected after 7 days of culture, observed as cells with many small prolongations. In the experimental procedure with FGF8, this factor was added to the culture using the same differentiation medium (Figure 1D). Independently of the treatment, the cultures were mainly composed of oligodendrocytes, as analyzed by Olig2 staining (Figure 1E–H). In the cultures with FGF8, an increased number of cells were detected compared to the other treatments, despite performing the cultures with the same initial number of cells. To corroborate that FGF8 induced proliferation, BrdU was incorporated to the culture 24 hours before fixing, and then immunocytochemistry for BrdU and double-stained with NG2+ (an oligodendrocyte progenitor cell marker) to confirm that only oligodendrocytes were counted (Figure 1I–L). As a result, the oligodendrocyte progenitor cell cultures where FGF8 was added presented a marked increase in proliferation, even more so than in the undifferentiated cultures (Figure 1H). This confirmed that FGF8 induced the proliferation of OPCs.

### 2 FGF8 Upregulates the Expression of Early Oligodendrocyte Progenitor Cell Markers

**Figure 2:**
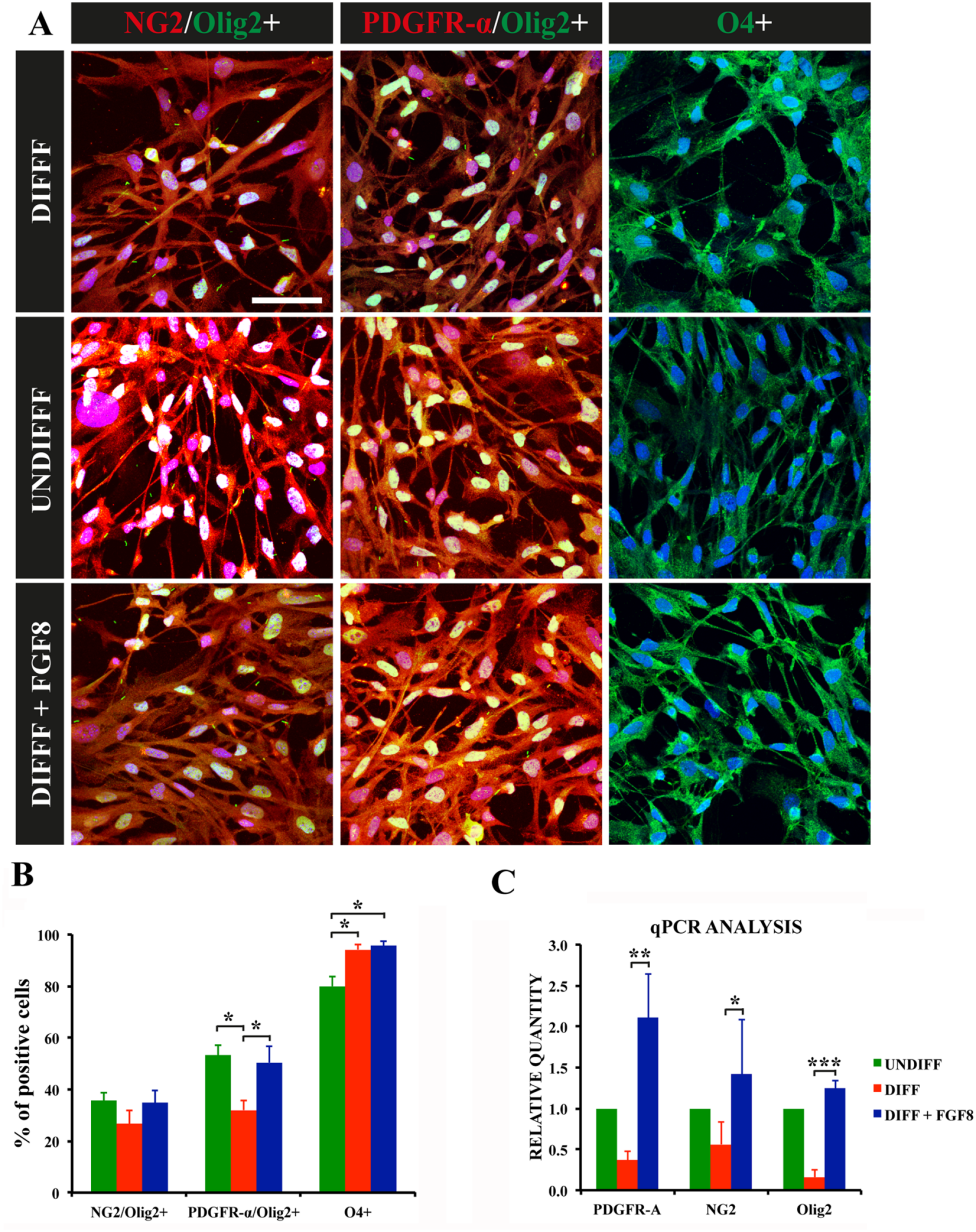
Oligodendrocyte progenitor cell differentiation with and without FGF8. A) Immunocytochemistry of NG2+/Olig2+, PDGFR-A+/Olig2+ and O4+ cells in the undifferentiated, differentiating and FGF8 culture media. In all images, blue staining corresponds to the nuclei (DAPI). Scale bar indicates 50 µm. B) Histogram depicting the percentage of cells in the different cell cultures staining for markers analyzed in A. Percentages are with respect to the total number of cells. C) Histogram depicting the results from the quantitative PCR analysis of the different cell cultures. *p<0.05, **p<0.01, ***p<0.001 (paired t-test in B, one way-ANOVA in C), n = 4.

As proliferation is enhanced, it is possible that differentiation may be hampered in turn. To confirm this, immature oligodendrocytic and oligodendrocyte progenitor cell markers NG2 and PDGFR-α, oligodendrocyte marker Olig2 and mature oligodendrocyte marker O4 were analyzed (Figure 2A). As a result, the percentage of cells positive for PDGFR-α and Olig2 was lower in the differentiated cultures, compared to the undifferentiated and FGF8 treatments (Figure 2B). As for O4, which begins to be expressed during oligodendrocyte maturation, coinciding with the loss of expression of NG2 and PDGFR-α [43], was increased in the differentiated and FGF8 treatments. This data was corroborated by QPCR (Figure 2C), indicating that the differentiated treatment resulted in less immature oligodendrocytes and OPCs, coinciding with more mature oligodendrocytes.

It is interesting to note that FGF8 treatment presented both more immature and mature markers. The composition of this culture medium was the same medium used for differentiation, except for the growth factor. Therefore, this data seems to indicate that FGF8 induces proliferation, resulting in more OPCs in culture, and does not hamper differentiation, which in turn results in more mature oligodendrocytes in the culture.

### 3 FGF8 Induces the Migration of Oligodendrocyte Progenitor Cells

**Figure 3:**
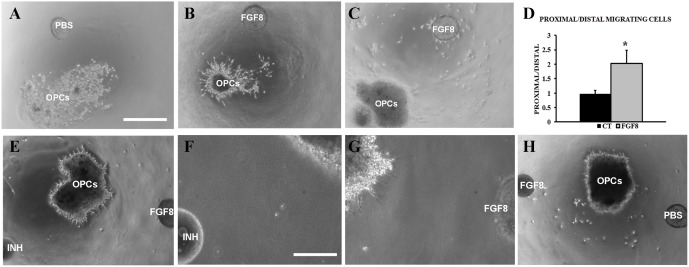
Matrigel cultures of OPCs. A) Oligodendrocyte progenitor cell matrigel culture where a PBS-soaked bead was placed 0.5 mm away from the cell cluster. Image taken at 40X magnification. B–C) OPCs where a cluster of cells migrating towards a FGF8-soaked bead can be observed. D) Histogram depicting the oligodendrocyte progenitor cell proximal/distal rate in control cultures and where FGF8 was included. *p<0.01 (paired t-test, n = 4). (E–G) Matrigel cultures where on one side a FGF8-soaked bead was placed and on the other side a FGF8 inhibitor. (F, G) Close-up images of the areas near the inhibitor-soaked bead (F) or near the FGF8-soaked bead (G). H) OPCs where a FGF8-soaked bead was placed and on the other side a PBS-soaked bead, as control of the inhibitor. Scale bar indicates 500 µm for (A–C, D, H) and 200 µm for (F, G). All images were taken 48 hours after culture.

Undifferentiated OPCs were placed on matrigel cultures to perform migration assays. To this end, a heparin bead previously soaked in FGF8 solution (500 ng/ml) was placed 0.5 mm away from the cluster of OPCs, while a PBS-soaked bead was used as control (Figure 3A–C). Twenty four hours afterwards, outgrowths were observed from the OPCs, the majority of which were detected in the direction of the FGF8-soaked bead (Figure 3B). The cultures were maintained and observed after 48 and 72 hours, with similar results (Figure 3C), the OPCs reaching the heparin bead. To confirm the attracting effect of FGF8, proximal/distal analysis was performed on the cultures (Figure 3D). The cultures where FGF8-soaked beads were used presented a significantly higher proximal/distal ratio than control cultures where PBS was used. Specifically, a 2-fold increase in cells was detected near the beads than in distal areas of the cell clusters. Therefore, the results indicated a predominantly migrating, attracting effect induced by FGF8.

In order to confirm this migrating property, the FGF-receptor inhibitor SU5402 was used in the cultures to counteract the effect of FGF8 (Figure 3E–G). In this case, two beads were used, on one side of the cluster of OPCs, a FGF8-soaked bead was placed, while on the other end a bead soaked in the inhibitor. As control of the inhibitor a PBS-soaked bead was used (Figure 3H). As a result, after 48 hours of culture, in the side of the OPCs where the SU5402-soaked bead was placed, no cell sprouting or migration was detected (Figure 3F). In the other side of the cluster, nearest the FGF8-soaked bead, numerous cell outgrowths were present (Figure 3G), indicating that the OPCs were stimulated by the FGF8 but the inhibitor managed to halt further migration. In the cases where PBS was used instead of the inhibitor, migration of the OPCs was observed as in the previous results (Figure 3H).

Overall, the results demonstrated that FGF8 is capable of inducing oligodendrocyte progenitor cell migration, exerting and attracting effect, and is mediated by the binding of FGF8 to FGF receptors.

### 4 Transplantation of FGF8-Soaked Beads Activates the Migration of Oligodendrocyte Progenitor Cells In Vivo, Exerting an Attracting Effect

**Figure 4:**
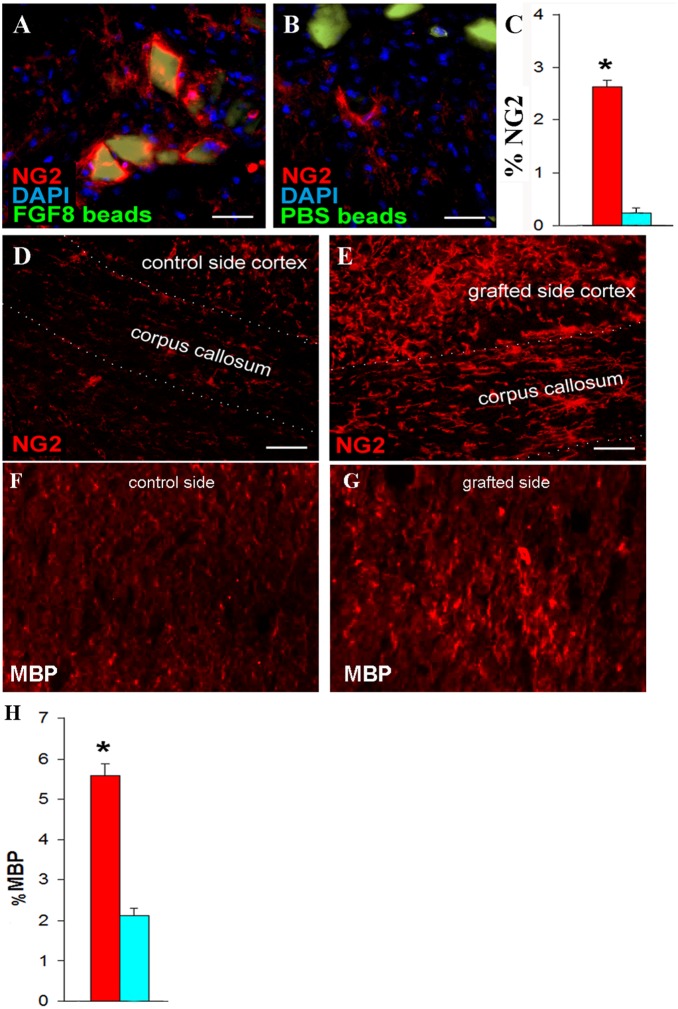
In Vivo Transplantation of FGF8-soaked beads in Chronically Demyelinated Mice. A) FGF8-soaked heparin beads (seen in green) surrounded by NG2-positive cells (in red. B) Control experiment where PBS-soaked beads were transplanted. C) Histogram depicting the percentage of NG2 immunoreactivity in the area injected with the heparin beads. The red bar indicates the percentage in the case FGF8-soaked beads was used, while the blue bar indicates the sham control. D) NG2 immunoreactivity in the corpus callsoum and cortex in the area near the PBS-soaked beads. E) NG2 immunoreactivity in the corpus callsoum and cortex in the area near the FGF8-soaked beads. F, G) MBP immunoreactivity in the area near the PBS or FGF8-soaked beads, respectively. H) Histogram depicting the percentage of MBP immunoreactivity in the area injected with the PBS- (blue bar) or FGF8- (red bar) soaked heparin beads. Scale bar measures 50 µm. *p<0.01 (paired t-test), n = 4.

In order to confirm if the results observed in vitro can be extrapolated in vivo, as well as analyze if FGF8 may be used as a therapeutic tool in inducing remyelination, mice were treated with cuprizone for 12 weeks to induce a chronic demyelinating condition, similar to previous studies [13], [44], [45]. Then, FGF8-soaked heparin beads were grafted on one side of the fimbria closest to the corpus callosum, while in the other side a PBS-soaked bead was grafted. As a result, 1 month after transplantation, an increase in NG2 positive cells was detected in the area where the beads were transplanted, some of which were surrounding the beads (Figure 4A). The side where PBS-soaked beads were used, on the other hand, presented lower NG2 immunoreactivity in the area compared to the group where FGF8-soaked beads were grafted (Figure 4B, C).

Also, both NG2 and MBP (oligodendrocyte progenitor cell and mature oligodendrocyte markers, respectively) immunoreactivity were detected in the corpus callosum near the area where the FGF8-soaked beads were grafted, which was not detected in the side where PBS-soaked beads were used (Figure 4D–H). Thus, FGF8-mediated stimulus was capable of reaching the corpus callosum from the fimbria.

## Discussion

In this work, we have proven that FGF8 is capable of activating post-natal mouse oligodendrocyte progenitor cells (OPCs) both in vitro and in vivo. Proliferation and migration of OPCs, as well as their differentiation to mature oligodendrocytes was also observed in vitro, indicating that this process is not hampered.

Oligodendrocytes are known to express various FGF receptors, mainly several subtypes of FGFR1, FGFR2 and FGFR3 [46]. Many works have focused on the study of the expression of these receptors, as well as the effect FGFs have on the cells, which varies throughout its lineage. For example, FGF2, when combined with PDGF-AA, is known to induce proliferation in OPCs [47], [48], while mature oligodendrocytes in the presence of this growth factor revert to a progenitor state [49], [50]. This indicates a negative effect in terms of differentiation and myelin production using FGF2. On the other hand, another work has shown that OPCs cultured in the presence of FGF8 expressed significantly more MBP compared to FGF2 [51], revealing a distinct effect of these two at-first similar growth factors. This same article also demonstrated that in oligodendrocyte cultures, while FGF2 downregulated mature oligodendrocyte markers and induced a reverted state, this was not observed with FGF8. In our work, we have further demonstrated FGF8 is not only capable of inducing a proliferative effect on OPCs in culture, but is also capable of attracting these cells and allowing their differentiation to myelinating oligodendrocytes. Thus, while FGF2 is useful in inducing OPC proliferation at the handicap of blocking differentiation, FGF8 seems to possess similar properties without this impediment.

The mouse model used in this study, where cuprizone was added to the micés diet, results in a chronic, irreversible demyelinating model similar to the lesions observed in multiple sclerosis. If the cuprizone is removed prematurely (before the 12 weeks), remyelination is spontaneously activated, which occurs in two phases: OPCs proliferation and colonization of the demyelinated area, and differentiation towards immature oligodendrocytes that bind to the demyelinated axons and mature to myelinating oligodendrocytes. This process is regulated by the coordinated expression of soluble factors [52].

The remyelinating process observed by early cuprizone removal also occurs naturally in vivo. OPCs are expressed throughout the adult central nervous system, and are capable of differentiating to mature oligodendrocytes when a demyelinating lesion occurs [6]. However, in the chronic phase of multiple sclerosis, there is a multiple dysfunction in the activation of OPCs, which affects the remyelinating process [53]. This may be due to either an absence of nearby OPCs or that they are quiescent and cannot react. Regardless of the reason, the lack of soluble factors that activate the migration and differentiation of OPCs may be one of the primary reasons for this failed response. Thus, for a therapeutic approach to be effective it must be capable of stimulating the quiescent OPCs, as well as induce their migration to the damaged area. This work demonstrates that FGF8 may be used to this end.

In conclusion, FGF8 is a morphogenetic factor that may be used to induce post-natal migration, proliferation and differentiation of oligodendrocyte progenitor cells. This property makes it a candidate factor that may be used in demyelinating disorders.
